# Autoimmune/Inflammatory Syndrome Induced by Adjuvants (ASIA) in a Patient With Silicone Breast Implants and Scleroderma-Like Manifestations: A Case Report

**DOI:** 10.7759/cureus.108552

**Published:** 2026-05-09

**Authors:** Beatriz Soto-Cala, Sofia Restrepo-Samper, Claudia Gonzalez, Daniel S Perdomo-Pachon, Juan G Chalela

**Affiliations:** 1 Medicine, Sofia Restrepo-Samper's Private Dermatology Clinic, Bogotá, COL; 2 Dermatology, Universidad Militar Nueva Granada, Bogotá, COL; 3 Radiology, Universidad del Rosario, Bogotá, COL; 4 Dermatology and Internal Medicine, Fundación Santa Fe de Bogotá, Bogotá, COL

**Keywords:** autoimmune response, breast implants, clinical case report, foreign-body granuloma, immunologic adjuvants, limited systemic sclerosis

## Abstract

Autoimmune/inflammatory syndrome induced by adjuvants (ASIA) encompasses a spectrum of immune-mediated clinical conditions triggered by exposure to adjuvant substances such as aluminum, vaccine components, and silicone breast implants (SBIs).

We present the case of a 68-year-old woman with a history of silicone breast augmentation, followed by implant rupture and reimplantation. Years later, she developed Raynaud's phenomenon, cutaneous calcinosis, and sicca symptoms. The patient also exhibited cognitive impairment and depressive symptoms. Laboratory investigations confirmed the presence of anti-centromere antibodies and elevated acute-phase reactants. Nailfold capillaroscopy demonstrated an early scleroderma pattern. Imaging revealed silicone migration into axillary lymph nodes, histopathologically consistent with siliconomas. A diagnosis of limited cutaneous systemic sclerosis was made. Following implant removal and the excision of silicone-laden lymph nodes, the patient experienced significant improvement in systemic, cutaneous, and neuropsychiatric symptoms, with subsequent normalization of her autoimmune profile, supporting the diagnosis of ASIA.

While the relationship between SBIs and systemic autoimmune manifestations remains controversial, this case highlights the broad clinical spectrum of ASIA and underscores the importance of clinical suspicion, multidisciplinary evaluation, and individualized therapeutic strategies in patients with unexplained autoimmune manifestations and a history of adjuvant exposure.

## Introduction

Since the 1960s, silicone breast implants (SBIs) have been increasingly used, and augmentation mammoplasties remain among the most commonly performed cosmetic procedures worldwide. In 2023, 1.8 million of these surgeries were performed, including 28,000 in Colombia alone, which accounted for 27% of all cosmetic surgical procedures in that country [1]. Despite their widespread use, SBIs are increasingly recognized as immunogenic adjuvant devices capable of inducing autoimmune-like phenomena [2].

Adjuvants, including silicone, squalene, hyaluronic acid, mineral oils, vaccines, metals, and other environmental agents, are compounds that can enhance a specific immune reaction once introduced into the body, resulting in a rise in antibodies against specific pathogens [2,3]. In 1964, Miyoshi et al. were the first to introduce the term "human adjuvant disease" when describing patients with nonspecific symptoms following silicone or paraffin exposure, as later summarized by Alijotas-Reig [4]. It was not until 2011 that Shoenfeld and Agmon-Levin introduced the term "ASIA" (autoimmune/inflammatory syndrome induced by adjuvants), a complex disorder encompassing various medical conditions associated with exposure to several adjuvant substances that trigger aberrant immune responses and promote the development of autoantibodies [5]. Between 2011 and 2016, over 4,000 cases of ASIA were identified, with more than 90% occurring in women. 

In the dermatological context, the relevance of ASIA stems from its association with both systemic and cutaneous manifestations, which may serve as early indicators of an exaggerated immune response. These include chronic fatigue, sleep disturbances, dementia, arthralgias, lupus-like skin lesions, scleroderma-like features, vasculitis, and Raynaud's phenomenon [2,3]. Clinical manifestations are associated with abnormal laboratory studies, such as elevated acute-phase reactants and positive autoantibodies [4-6]. 

The relationship between SBIs and systemic autoimmune manifestations remains controversial, particularly since the widespread litigation that emerged in the United States during the 1990s attributing autoimmune conditions to silicone exposure without robust scientific data [7-9]. As a result, significant skepticism persists within the medical community. In this context, ASIA should be approached cautiously and considered a diagnosis of exclusion, particularly given the potential for misattribution of nonspecific symptoms.

In this report, we present a case of ASIA associated with SBIs in which the patient developed clinical features of limited cutaneous systemic sclerosis, progressive neuropsychiatric decline, and significant symptomatic improvement following explantation.

This case was previously presented as an oral presentation at the XXXI Colombian Congress of Dermatology, Medellín, Colombia, on April 26, 2025.

## Case presentation

A 68-year-old woman presented to the dermatologist complaining of hair loss, trichodynia, scalp pruritus, and firm nodular lesions on her elbows and knees for the past few months. Additionally, she reported fatigue, myalgias, sleep disturbances, dizziness, mucosal dryness, dysphagia, cough, color and morphologic changes in her fingers, diarrhea, and involuntary weight loss during this period. Her medical history included gastroesophageal reflux disease, and she denied any personal or family history of autoimmune disease. She had received augmentation mammoplasty with silicone implants 20 years earlier. Following an incidental implant rupture with secondary siliconomas, she underwent explantation and reimplantation with new prostheses two and three years prior to symptom onset, respectively.

**Figure 1:**
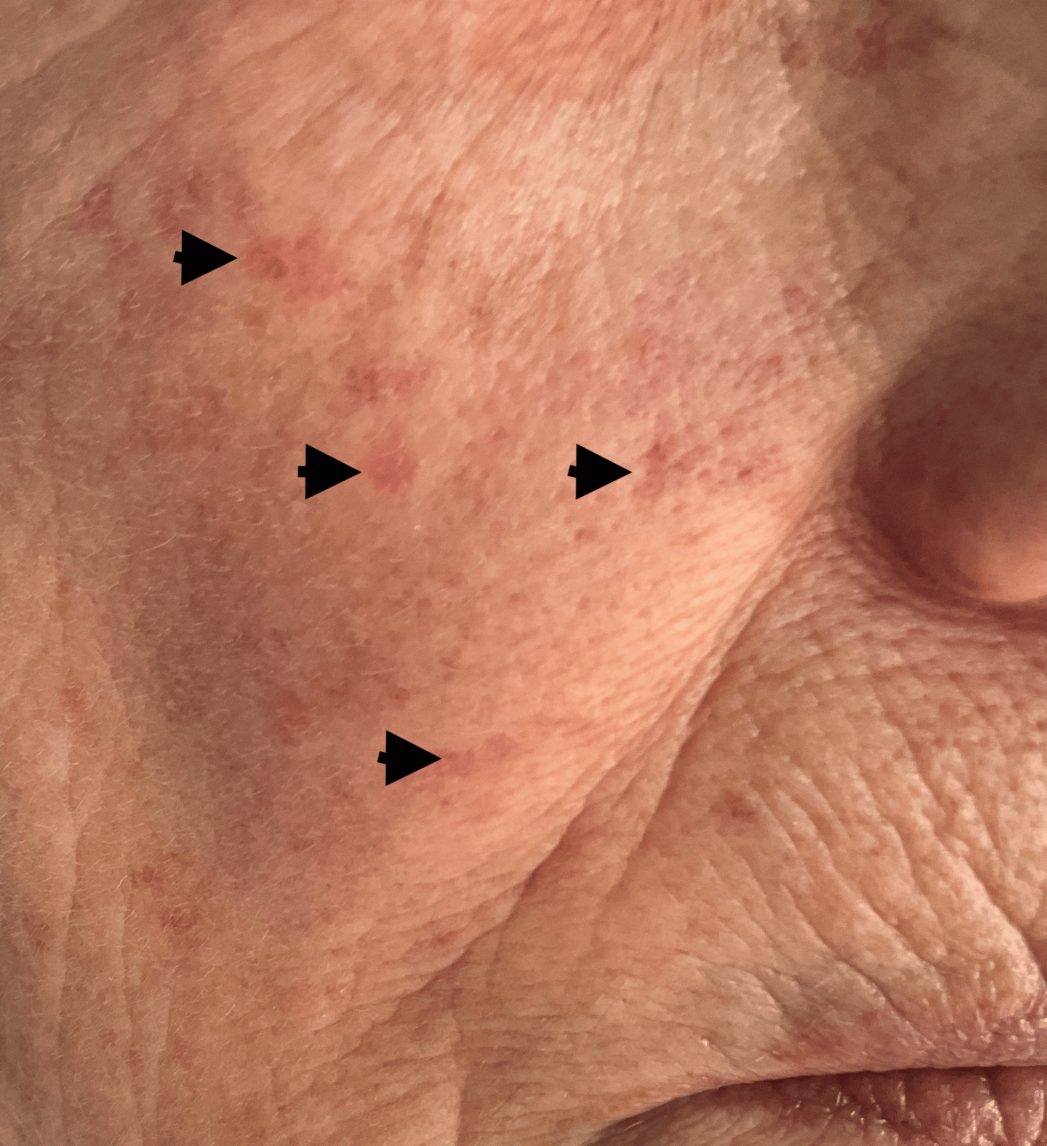
Palpebral erythema with associated fine telangiectatic vessels visible on the malar region

**Figure 2:**
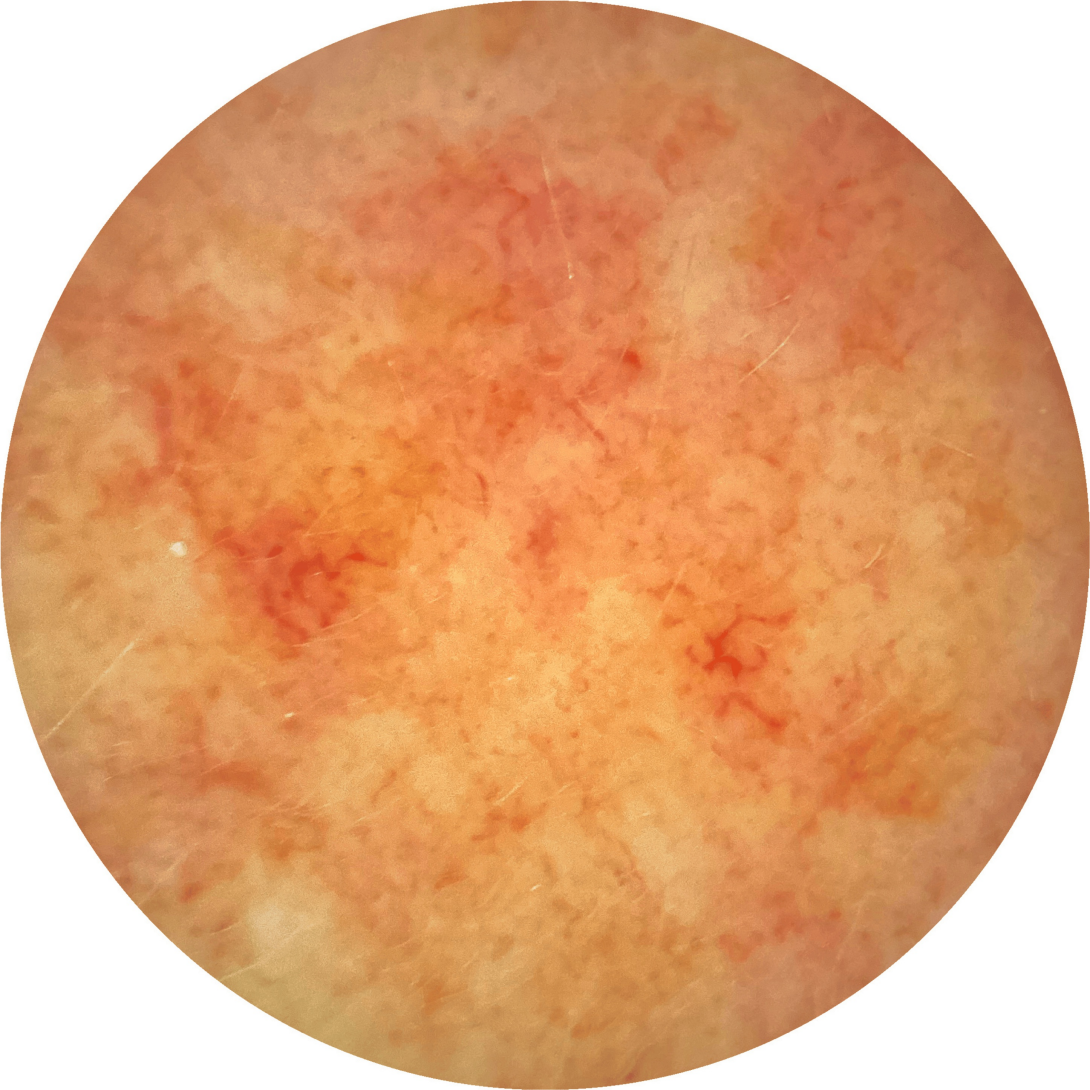
Dermatoscopic view of the prominent telangiectasias over the malar areas, consistent with vascular changes classically present in autoimmune and/or connective tissue disease

**Figure 3:**
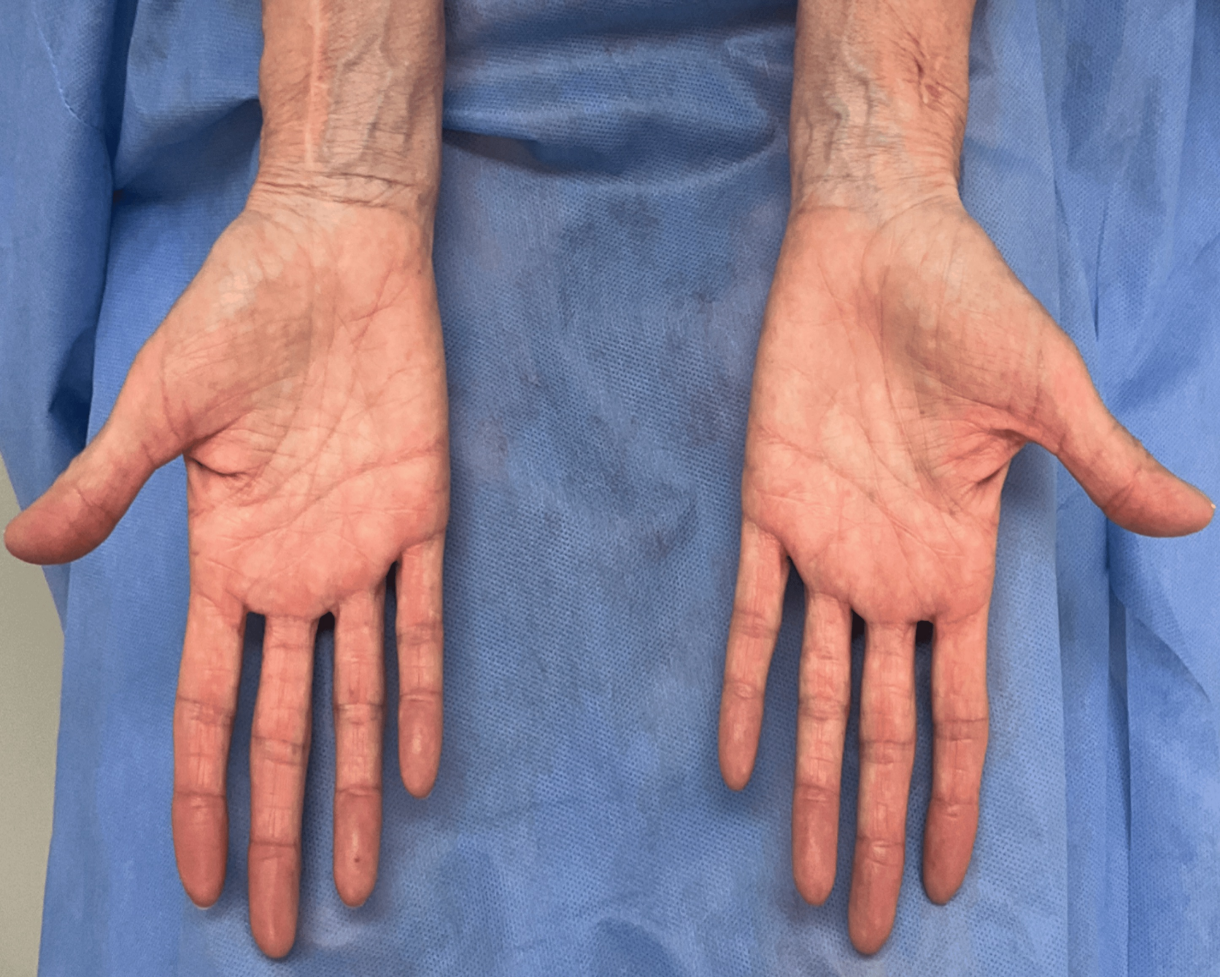
Palms and digits demonstrating skin thickening and petechiae

**Figure 4:**
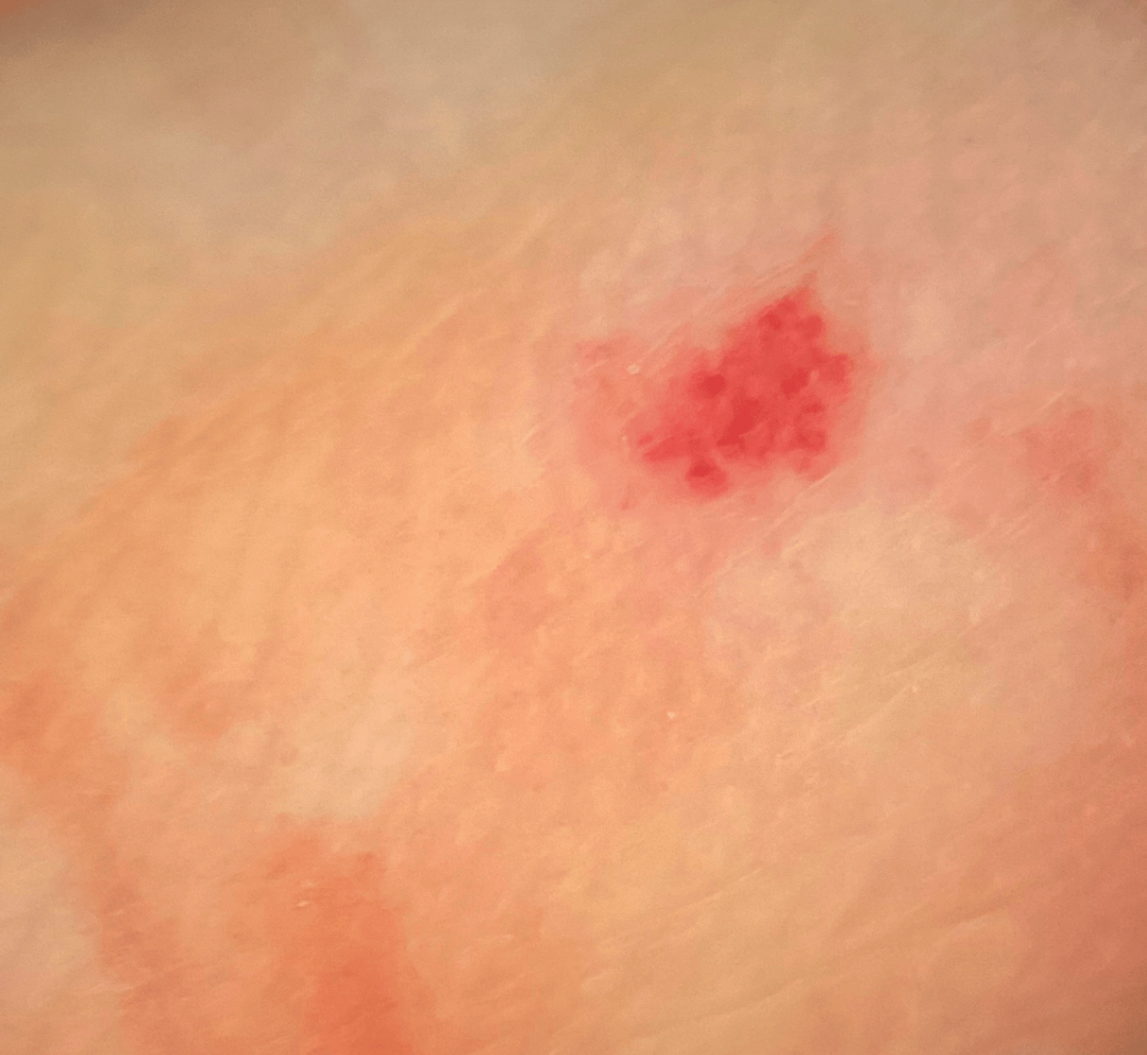
Dermatoscopic view of petechiae on palms

Physical exam revealed palpebral erythema, malar telangiectasias (Figures 1-2), firm nodules on elbows and knees, petechiae on hands and face, and digital skin thickening (Figures 3-4). Scalp examination and trichoscopy suggested diffuse alopecia.

Laboratory studies demonstrated positive antinuclear, anticentromere (1/160), and anti-PM/Scl antibodies. The remainder of the autoimmune profile was negative. Acute-phase reactants were elevated. Renal, metabolic, and hepatic function were normal. Vitamin D and complement levels were also normal (Table 1). 

**Table 1:** Laboratory studies at symptom onset

Test	Result	Reference range
Autoimmune profile		
Antinuclear antibodies (ANAs)	1:640 nucleolar pattern, later confirmed by a titer of 1:2560, homogeneous pattern	Negative
Anticentromere antibodies	1:160	<1:80
Anti-PM/Scl antibodies	Positive	Negative
Anti-Scl-70 antibodies	Negative	Negative
Anti-DNA antibodies	Negative	Negative
Anti-Jo-1 antibodies	Negative	Negative
Anti-PL-7 antibodies	Negative	Negative
Anti-PL-12 antibodies	Negative	Negative
Anti-SRP54 antibodies	Negative	Negative
Anti-Mi-2 antibodies	Negative	Negative
Anti-Ku antibodies	Negative	Negative
C3	89 mg/dL	85-161 mg/dL
C4	23 mg/dL	11-44 mg/dL
Acute-phase reactants		
C-reactive protein (CRP)	23 mg/L	<3 mg/L
Erythrocyte sedimentation rate (ESR)	42 mm/h	Females over 50 years old: ≤30 mm/h

Nailfold capillaroscopy revealed an early scleroderma pattern characterized by isolated giant capillaries, few microhemorrhages, and a relatively preserved capillary distribution. Calcinosis cutis was confirmed histopathologically after a biopsy of a nodule in the elbow was taken; no radiographic imaging was performed. The diagnosis of limited cutaneous systemic sclerosis (formerly known as CREST syndrome) was made based on the presence of key clinical features, including calcinosis cutis confirmed by histopathology, Raynaud's phenomenon, esophageal involvement (dysphagia and gastroesophageal reflux), sclerodactyly evidenced by digital skin thickening, and telangiectasias on physical examination. In addition, serological testing and nailfold capillaroscopy findings further supported the diagnosis.

**Figure 5:**
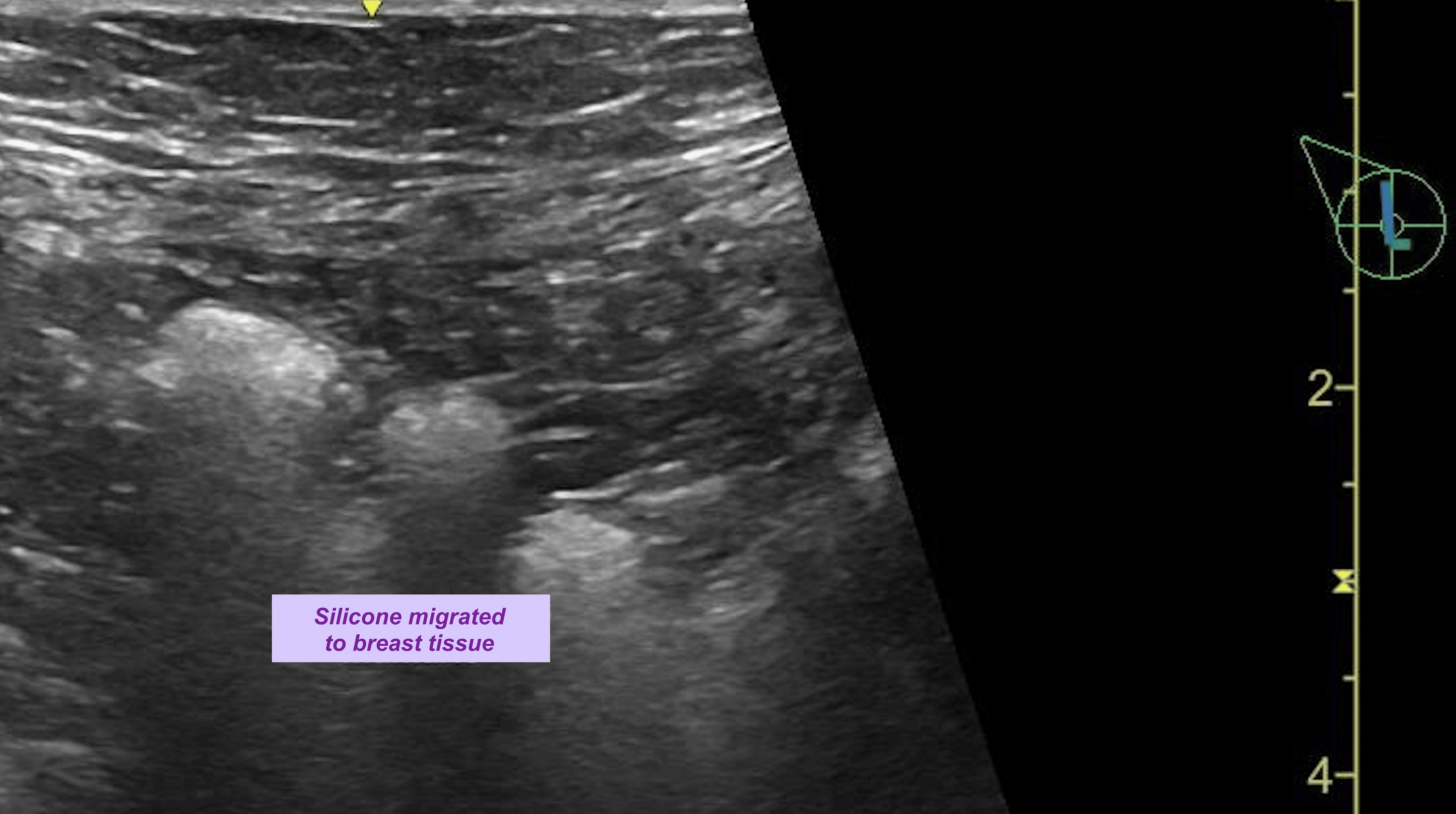
Breast ultrasound demonstrating the characteristic "snowstorm" appearance adjacent to the right implant, indicative of extracapsular silicone leakage

**Figure 6:**
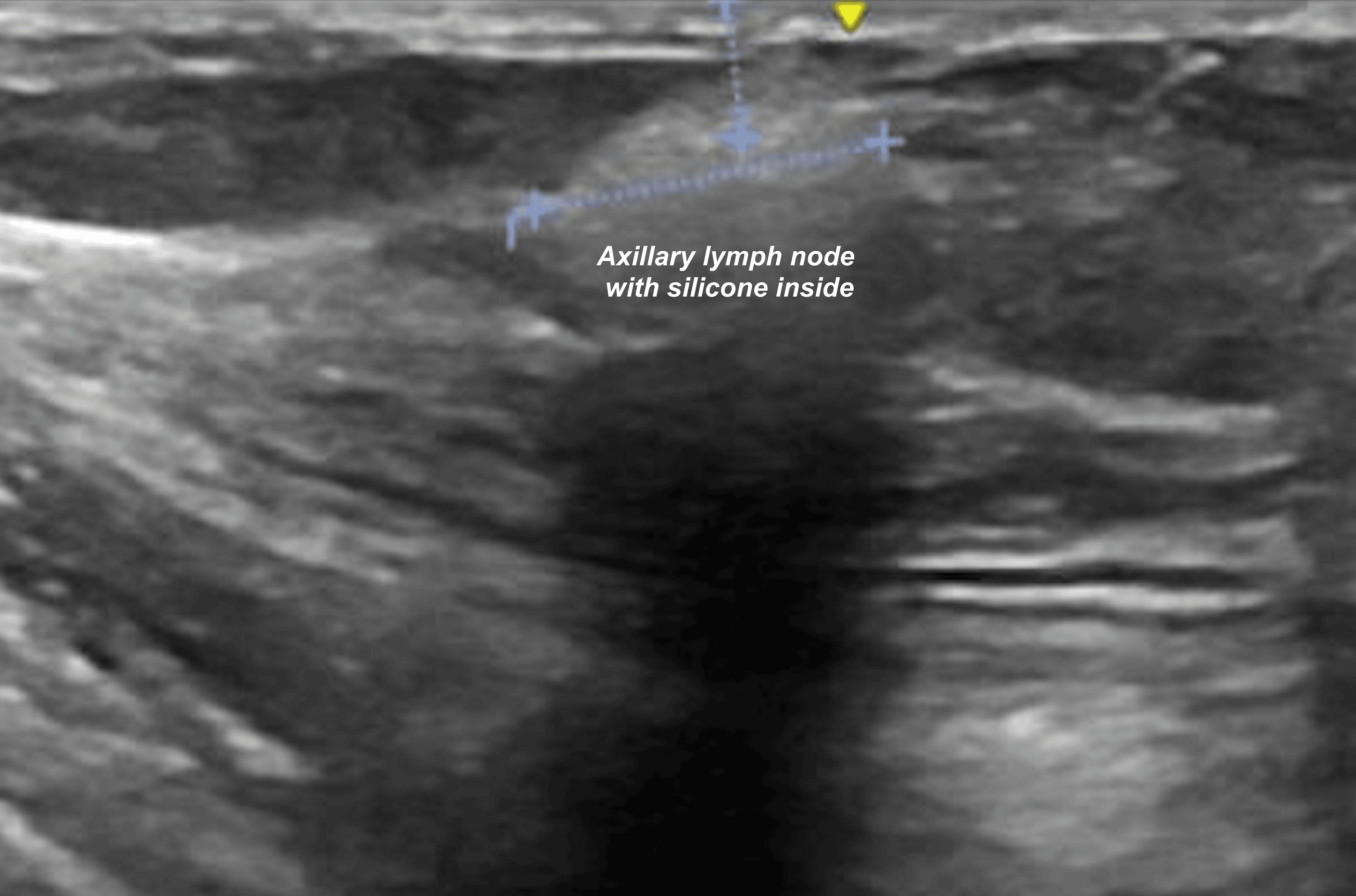
Ultrasound of the right axilla showing lymph nodes containing hyperechoic material consistent with silicone deposition

**Figure 7:**
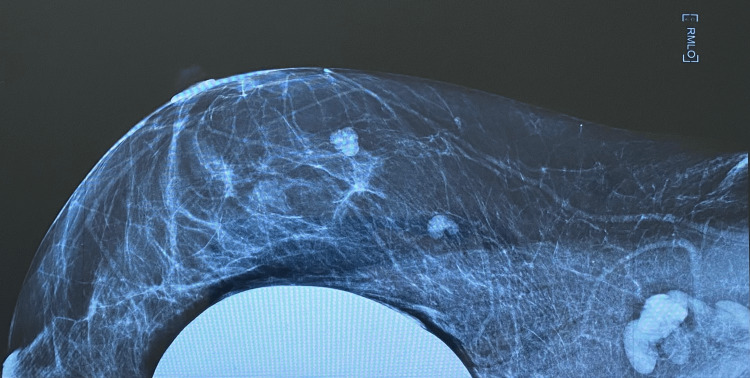
Mammographic view revealing dense, radiopaque nodules in the breast and axillary regions, compatible with silicone accumulation

For the next few years, she was followed by a multidisciplinary team including dermatology, rheumatology, gastroenterology, and otolaryngology. Four years later, she developed an important neurocognitive decline (memory loss, attention deficits), worsening of insomnia, and symptoms of severe anxiety and depression. Physical examination revealed palpable right axillary nodules. Breast ultrasound identified hyperechoic images described as a "snowstorm" pattern (Figure 5), as well as axillary lymph nodes occupied by silicone that had migrated into them (Figure 6). Mammograms also reported radiopaque nodules in the breast and axillary nodes (Figure 7). 

Given those findings, she underwent breast implant removal as well as excision of axillary nodes. Pathology studies were compatible with siliconomas. A few months later, she presented a significant improvement in her cutaneous, neuropsychiatric, and systemic symptomatology, and the treating multidisciplinary team considered that she fulfilled the diagnostic criteria for ASIA. One year later, the patient returned to the dermatologist for a follow-up evaluation with complete resolution of neuropsychiatric symptoms, a significant slowing down of her disease progression (improvement of digital skin thickening and color and temperature changes, absence of new telangiectasias), and normalization of her autoimmune profile (negative antinuclear, anticentromere, and anti-PM/Scl autoantibodies). No significant change in calcinosis was observed despite improvement in other manifestations.

## Discussion

Overview

Although widely used, SBIs have been associated with autoimmune-like phenomena [2]: they have been associated with a higher prevalence of autoimmune/autoinflammatory diseases, with an odds ratio (OR) of 1.22 (1.18-1.26) [1,10,11]. ASIA, first described by Shoenfeld and Agmon-Levin in 2011, encompasses a spectrum of conditions associated with exposure to adjuvant substances that trigger aberrant immune responses and promote the development of autoantibodies [5]. ASIA includes vaccination-induced autoimmune disorders, sarcoidosis, Gulf War Syndrome (GWS), macrophagic myofasciitis (MMF) with chronic fatigue syndrome, sick-building syndrome (SBS), Sjögren syndrome (SS), undifferentiated connective tissue disease (UCTD), silicosis, and silicone implant incompatibility syndrome [5,12]. Clearly identifiable clinical conditions are present in up to 89% of ASIA patients: the most diagnosed disorder was UCTD, followed by fibromyalgia and/or chronic fatigue syndrome. Additional autoimmune diseases identified included systemic lupus erythematosus (SLE), various forms of vasculitis, and cutaneous sarcoidosis [3]. 

In their article, Shoenfeld et al. also mentioned a relatively new entity termed "siliconosis" encompassing symptoms secondary to silicone implants, either ruptured or intact, such as chronic fatigue, impaired cognition, depression, dry eyes and mouth, skin abnormalities, swollen axillary glands, hair loss, and headache [2,3], mostly present in our patient. Furthermore, silicone migration throughout the body via soft tissues, lymphatic vessels, or blood vessels may generate silicone-induced granulomas secondary to a chronic inflammatory reaction aimed at isolating silicone, also known as "siliconomas". They may be asymptomatic or present as nodules, masses, ulcers, or fistulas, potentially contributing to systemic immune dysregulation [13].

In ASIA secondary to SBIs, some studies have reported that Raynaud's phenomenon was the most frequently reported manifestation, followed by sicca symptoms. The most frequent conditions in SBI users are SS, UCTD, autoimmune thyroiditis, adult-onset Still's disease (AOSD), and systemic sclerosis [5]. Nailfold capillaroscopy findings suggested a potential association with scleroderma in certain cases. Moreover, elevated acute-phase reactants, positive antinuclear antibodies (ANAs), and autoantibodies linked to SS were commonly observed in this population [6]. Our patient did not fulfill the classification criteria for SS but did present clinical features of limited cutaneous systemic sclerosis. A separate analysis reported a notably high ANA positivity rate, approximately 58%, in women with SBIs [5]. 

Nonetheless, it is important to clarify that a causal association has not yet been definitively proven and that the current evidence is limited in both quantity and quality [14]. Besides, there is controversy around the high risk of bias due to self-reported nonspecific symptoms of this condition and inadequate long-term follow-up [5]. Indeed, the medico-legal context surrounding breast implants during the 1990s in the United States led to the perception of a causal relationship between breast implants and systemic symptoms [7]. However, these conclusions were often reached in the absence of robust scientific evidence; subsequent epidemiological studies have not consistently demonstrated a causal relationship, and methodological limitations in earlier studies have been widely recognized [7-9]. Attributing heterogeneous and nonspecific symptoms to adjuvant exposure may obscure alternative diagnoses and potentially delay the identification of well-defined autoimmune or systemic conditions. Thus, careful clinical evaluation and a balanced interpretation of the available evidence are required prior to diagnosing ASIA.

Pathogenesis

As mentioned above, ASIA is a group of immune-mediated conditions characterized by a complex interaction between an adjuvant, the immune system, and genetic factors. Chronic exposure to the adjuvant leads to a progressive liberation of the antigen that constantly stimulates the immune system [5]. This generates antibody production, blocks their elimination, and leads to a prolonged exposure to antigen-presenting cells (APCs). The antigen is then translocated to lymphatic ganglia, where it is recognized by T lymphocytes and converted from a soluble to a particulate form, which is phagocytized by APCs like macrophages, dendritic cells (DCs), and B cells. It is believed that B cells and DCs collaborate to induce the TFH1 and Th1 responses by the secretion of several cytokines, including interferon (IFN)-gamma and interleukin (IL)-12. There is a stimulation of Toll-like (TLR) and nucleotide-binding oligomerization (NOD) receptors, including the inflammasome NLRP3 [2]. Stimulation of NOD1, NOD2, and NLRP3 results in the secretion of more proinflammatory cytokines, including IL-1β and IL-18, which contribute to the differentiation of naïve T cells toward a Th2 phenotype [5], with subsequent IL-4 and IgE production [2].

Moreover, a localized immune response characterized by regulatory T cell (Treg) suppression and enhanced activation of Th1 and Th17 subsets may foster a proinflammatory milieu, promoting cytokine release and sustained inflammation. Additionally, reactive oxygen and nitrogen species are generated, promoting macrophage apoptosis. This process releases particulate matter, including silicone, which undergoes oxidization within the body and becomes silica, which later induces a marked increase in tumor necrosis factor (TNF)-alpha, IL-6, and IL-17 production, contributing to neutrophil activation, enhanced reactive oxygen species (ROS) generation, and the release of enzymes such as myeloperoxidase [5] or proinflammatory markers like C-reactive protein [12]. Thereby, silicone generates a foreign body-like reaction, initially as an acute inflammatory response and afterwards as a chronic fibrotic response, leading to the development of an autoimmune systemic disease [11,12]. Ultimately, this hyperstimulated adaptive immune response may even contribute to the development of malignancies such as non-Hodgkin lymphomas [3,14].

Besides, while the exact pathogenetic mechanism is still not fully understood, the Janus kinase transcriptional signal transducer activator (JAK/STAT) pathway may also play a role in the pathogenesis of ASIA, as observed in other autoinflammatory and autoimmune diseases [5].

Finally, it has been proven that genetic predisposition can lead to signs and symptoms of systemic autoimmunity following environmental exposure to adjuvants [11]. In particular, ASIA has been associated with human leukocyte antigens, such as HLA-B8, HLA-DRB1, HLA-B27, HLA-DR3, and HLA-DQB1, or specific haplotype combinations [2,10]. Mutations in genes like protein tyrosine phosphatase non-receptor type 22 (PTPN22), which regulates both T and B cells, have also been associated with various autoimmune diseases [5]. It is believed that those HLA genes predispose to a loss of immunologic tolerance to antigens, leading to an overactivation of the immune system.

In our patient's case, it is worth noting that she had no personal or family history of autoimmune disease; however, she did not undergo any genetic study, so it cannot yet be determined whether ASIA was purely secondary to non-genetic factors. Due to the notable environmental exposure and the relatively rare occurrence of ASIA, researchers have proposed a potential role for epigenetic mechanisms in its pathogenesis [5].

Diagnostic criteria

ASIA is typically diagnosed after excluding alternative conditions [12]. In 2011, Shoenfeld and Agmon-Levin proposed 12 diagnostic criteria for ASIA: two major criteria OR one major PLUS two minor criteria are required to establish the diagnosis [2,12]. However, they have been strongly criticized for their low specificity since they include manifestations that can correspond to a broad range of conditions. In response to those critics, they were modified by Alijotas-Reig to make them more objective and clinically relevant [4], but these criteria have not been formally validated yet. In the following table, we present the modified diagnostic criteria, as well as those fulfilled by our patient.

**Table 2:** Diagnostic criteria for autoimmune/inflammatory syndrome induced by adjuvants and criteria met by our patient SBI: silicone breast implants; HLA: human leukocyte antigen; ACE: angiotensin-converting enzyme; LDH: lactate dehydrogenase

Criterion	Description	Our patient
Major criteria		
Exposure to a potential trigger	Prior contact with adjuvants (silica, squalene, hyaluronic acid, mineral oils, vaccines, metals) before the onset of symptoms	Exposure to SBIs
Latency period	A minimum interval must pass between exposure and symptom onset (at least several days for vaccines and at least one month when implants or other biomaterials are involved)	Symptoms began two decades after SBI surgery and three years after implant rupture
Clinical manifestations	Local manifestations: inflammatory nodules, edema or angioedema, indurated skin, pseudocysts or abscesses, enlarged lymph nodes, panniculitis, morphea-like lesions, sarcoid-like lesions	Local: nodules on knees and elbows
	Systemic manifestations: remote inflammatory nodules, arthritis, sicca symptoms, Sjögren's syndrome, myositis or muscle weakness, widespread panniculitis, neurological involvement with demyelination, or evolution into autoimmune diseases	Systemic: sicca symptoms, Raynaud's phenomenon, sclerodactyly, telangiectasias
Histopathologic findings	Biopsy of affected tissue or lymph nodes showing a foreign-body reaction and/or findings demonstrating granulomatous/autoimmune disorders	Biopsy of axillary nodes compatible with siliconomas
Clinical improvement after removal of trigger	Symptoms improve or stabilize after the inciting adjuvant is removed	Removal of implants resulted in symptomatic improvement
HLA association	Specific HLA types (e.g., HLA B8, DRB1, DR3, DQB1) or certain haplotypes associated with autoimmune susceptibility	Molecular testing not performed
Minor criteria		
Recent provoking event	An aggravating factor or a new exposure occurring shortly before symptom onset	SBI rupture three years prior to the first clinical manifestations
Vascular skin changes	Sudden development of large areas of livedo reticularis and/or erythematous changes on the hands at disease onset	Raynaud's phenomenon
Abnormal laboratory studies	One or more of the following: autoantibody positivity, hypergammaglobulinemia, elevated ACE or LDH, decreased complement levels	Positive antinuclear, anti-PM/Scl, and anticentromere antibodies

Treatment

There are no standardized treatment guidelines that can be applied to all patients with ASIA [10]. While some articles suggest that the removal of breast prosthesis can improve functional symptoms in 50-98% of cases [3], this benefit is not universal and is consequently not routinely recommended [12,14]: other studies report that up to 25-40% of patients do not see any difference after SBI explantation, especially when it is not combined with immunosuppressive therapy [15]. The decision of removal may be challenging due to additional costs, surgical risks, and uncertainty about an actual benefit [1,10]. In non-surgical candidates or in patients with persistent symptomatology after removal, there is no evidence of a specific pharmacological treatment for ASIA; but in severe cases, experts agree that a systemic treatment should be administered. Some publications report successful treatments with systemic immunomodulators, such as corticosteroids at high doses, colchicine, cyclosporine, hydroxychloroquine, azathioprine, and/or cyclophosphamide [1,10,11].

Finally, it has been suggested that vitamin D deficiency increases the risk of autoimmunity in patients with implant-related disease (RR 3.14; 95% CI 1.24-7.95; p=0.009) [1]. Although evidence remains very limited, and despite vitamin D not representing a curative or standalone treatment for ASIA, considering its favorable risk-benefit profile, it may be reasonable to assess serum vitamin D levels prior to breast prosthesis implantations and initiate supplementation in cases of deficiency [16], with the aim of potentially reducing the risk of developing ASIA. Regardless of the treatment decision, it should be made by a multidisciplinary team, considering the clinical manifestations, the risk-benefit profile, and the patient's preferences.

In patients with limited cutaneous systemic sclerosis, there are currently no validated serological markers to reliably monitor disease progression. IL-6, a cytokine involved in the pathogenesis of systemic sclerosis and found in elevated concentrations in the skin and serum of patients with diffuse cutaneous systemic sclerosis, has been proposed as a potential marker for treatment response; however, supporting evidence remains limited [17,18]. Assessment of disease progression is primarily based on clinical evaluation, including pulmonary function tests, such as forced vital capacity (FVC) and diffusing capacity for carbon monoxide (DLCO), in cases with pulmonary involvement. Additional tools include physician-led clinical assessment and patient-reported outcome measures, such as the modified Rodnan skin score which quantifies skin thickness at various body areas [17] or the Health Assessment Questionnaire Disability Index (HAQ-DI). The latter was first developed for patients with rheumatoid arthritis and evaluates dressing and grooming, getting up, eating, walking, hygiene, reaching, grasping, and activities [17]. The optimal method for evaluating disease activity is still unclear, and novel composite measures are needed to accurately capture disease severity, progression, and objective improvement [18].

Clinical recommendations

Clinicians should maintain awareness of the potential association between SBIs and immune-mediated manifestations, particularly in individuals with pre-existing autoimmune conditions or prior reactions to adjuvants. Careful patient counseling before cosmetic procedures is advisable, including discussion of ASIA as a rare but potentially impactful condition that may affect quality of life.

## Conclusions

This case highlights the importance of considering ASIA in patients with unexplained autoimmune manifestations and a history of silicone breast implants. The presence of systemic, cutaneous, and neuropsychiatric symptoms, along with objective immunologic abnormalities, supported the diagnosis in our patient. Notably, significant clinical and serological improvement following implant removal reinforces the potential role of silicone as a trigger of immune dysregulation. Early recognition and a multidisciplinary approach are essential to optimize patient outcomes.
